# Enhancing the Management of Non-Specific Neck Pain through Gamification: Design and Efficacy of a Health Application

**DOI:** 10.3390/bioengineering11070640

**Published:** 2024-06-23

**Authors:** Yiran Sun, Yanjie Xian, Hongbo Lin, Xing Sun

**Affiliations:** Shenzhen International Graduate School, Tsinghua University, Shenzhen 518055, China; syr23@mails.tsinghua.edu.cn (Y.S.); xianyanjie@mails.tsinghua.edu.cn (Y.X.);

**Keywords:** gamification, serious games, optical motion capture, digital health application, non-specific neck pain, cervical spine exercise, chronic pain intervention, Neck Disability Index

## Abstract

Chronic non-specific neck pain (CNNP) poses a substantial health and economic burden in China. This study introduces a gamified motion-sensing health application framework to address the limitations of existing health applications. The gamified cervical spine somatic exercise application employs motion capture technology alongside the smartphone’s built-in sensors to simulate accurate somatic interactions. Controlled experiments and data analyses demonstrated that the application significantly outperformed traditional text and video interventions in relieving participants’ neck pain by increasing their average daily activity and compliance with the cervical spine exercise routine. The neck pain level of the participants is quantified by the Neck Disability Index (NDI). The results from the controlled experiments demonstrate that this gamified approach significantly decreases the Neck Disability Index (NDI) score from 1.54 to 1.24, highlighting its ability to alleviate neck pain and increase user compliance.

## 1. Introduction

Prolonged sedentary behavior and frequent head-down work habits commonly lead to weakened tension between cervical muscles and connecting ligaments, eventually causing cervical disk pathology [1]. This type of neck pain, arising without a specific cause, is defined as chronic non-specific neck pain (CNNP) [2]. Despite significant advancements in medical technology in recent decades, the prevalence of CNNP continues to be substantial. A study published in the *British Medical Journal* in 2020 indicated that between 1990 and 2017, the global incidence rate of neck pain was as high as 3551 per 100,000 people, with even higher rates in East Asia, ranging from 4800 to 5100 cases [3]. Furthermore, a study by Zeng Xinying et al. in 2016 revealed that low back and neck pain accounted for approximately 16.243 million years of healthy life lost due to disability (YLD), representing 12% of the total disease burden [4]. Globally, in 2020, neck pain affected 203 million people (95% uncertainty interval 163–253), and the number of people with neck pain is projected to increase by 32.5% (95% uncertainty interval 23.9–42.3) globally to 269 million by the year 2050 [5]. The effects of CNNP are persistent and complex, with recurrent neck pain in 19–37% of the cases [6]. The condition not only impacts the physical and mental health of patients but also poses a serious economic burden in terms of social productivity and remedial costs.

As society becomes increasingly aware of cervical spine health issues, there is a growing demand for effective prevention and intervention measures. In traditional cervical spine interventions, patients learn how to train through educational text and video instructions, but they are usually prone to injuries due to incorrect imitation of procedures, insufficient knowledge of risks, and inaccurate control of exercise quantity.

The development of modern technology has enabled health management through mobile devices. For instance, smart fitness devices and online medical services have become essential for daily health management. However, applications for cervical spine health that involve somatosensory interaction are yet fully explored in the market, particularly those incorporating gamification elements.

Gamification has proven to enhance user engagement and behavior modification. In the field of rehabilitation, gamified rehabilitation training using motion capture technology has been widely applied in adjuvant therapy and preventive healthcare of diseases [7] affecting wrists [8], walking abilities [9], and vision [10]. For example, a game-based pharmaceutical teaching application developed by Sera L and colleagues provides an entertaining and interactive environment to enhance learners’ ability to solve pharmaceutical problems [11]. Additionally, studies like those conducted by Schonauer C using Kinect-designed games for back and cervical spine rehabilitation [12] and by France et al. for VR games targeting lower back pain treatment [13] have validated the effectiveness of gamified rehabilitation schemes through clinical trials. Moreover, research by Althoff T and others has shown that gamified interventions can significantly increase physical activity levels and reduce sedentary time, indicating potential benefits for public health [14]. These studies demonstrate that, compared to traditional treatment methods, gamification is more effective at alleviating symptoms, providing greater control over the treatment process, and enhancing user compliance.

Based on these previous works, this study aims to explore the effects of gamified cervical spine exercise applications in the prevention and intervention of CNNP. The study employs an online survey distributed via WeChat, QQ spine health chat groups, and personal WeChat moments, to collect 273 users’ neck pain levels, treatment experiences, and expectations of health applications. The survey results showed that 158 respondents (58% of the total sample population) have mobile gaming habits and experience with health game applications. Nearly 60% of these respondents acknowledged the benefits of developing healthy habits through gamified applications.

Although the survey results indicated a high acceptance of gamified health applications among users and positive outcomes in other health management areas, the actual effectiveness in the prevention and intervention of CNNP remains unclear. Therefore, further research is required to validate the following question: *Can a gamified cervical spine somatic exercise application enhance the intervention effects on neck pain?*

Therefore, the study aims to evaluate the effects of two intervention methods on neck pain management: the traditional text and video interventions versus the gamified cervical spine somatic exercise application. This research proposes the following hypotheses:

**Hypothesis 1** **(H1).** *The gamification method is more effective than traditional text and video interventions for neck pain*.

The Neck Disability Index (NDI) [15] is an important parameter for quantifying cervical spine health status. The analysis will validate the relative effectiveness of gamified approaches for neck pain management via NDI ratings.

**Hypothesis 2** **(H2).** *The gamification method can enhance user compliance in neck pain interventions*.

The Technology Acceptance Model (TAM) is used to assess the use of information systems, which measures users’ acceptance of technology and its perceived usefulness [16]. The frequency of daily application use, the duration of the cervical spine exercise, and the data from the TAM are collected to evaluate user compliance. 

This study proposes a scientific framework for developing gamified somatosensory health applications. Following the framework, a mobile application for gamified cervical spine exercises is developed to promote cervical spine health exercise methods and enhance health awareness among mobile users, intervening in the adjunctive treatment of CNNP. 

## 2. Related Works

### 2.1. Review of Interventions for Chronic Non-Specific Neck Pain

Physical therapy is a common intervention, usually performed in clinical settings. Table 1 presents an array of common treatments for CNNP, offering succinct descriptions of each method. Pharmacological treatments (Drugs), despite their widespread use, have been documented to cause notable side effects and patient intolerance [17]. While physical therapy demonstrates efficacy in enhancing neck function and alleviating pain, it is often linked to higher costs and longer treatment durations [18].

Physical exercise is the simplest, most feasible, and cost-effective method among conventional treatments. This approach uses textual and video materials to provide health education, explaining the causes, prevention measures, and rehabilitation exercises for neck pain. These interventions aim to enhance patients’ knowledge and assist them in self-management. However, studies have shown that relying solely on this method often lacks the necessary depth and detail, potentially leading to misunderstandings or incomplete comprehension of complex medical information by the public [26].

In this study, rehabilitation exercises refer to a series of neck exercises that have been identified and selected. Price et al. [27] conducted a meta-analysis on the efficacy and optimal exercise dosage for various exercise training methods in treating CNNP. They reviewed 3990 related papers, encompassing a sample of 2288 subjects, and screened out 26 papers that met their research criteria. The study further extracted cervical spine exercise paradigms that could be gamified [28,29,30,31,32,33,34,35,36,37,38,39,40,41,42], the selected exercises are listed in Table 2 (all exercises have been proven to have clinical efficacy).

### 2.2. Theoretical Models of Healthy Game Design and Application

#### 2.2.1. Game for Health (G4H) Theory

In Baranowski T’s G4H study [44], several critical components for health game design were identified: game design and gameplay mechanics, behavioral determinants, behavioral interventions, identification of antecedents to health problems, and achievement of health goals. Unlike traditional video games designed primarily for entertainment, games developed under the G4H theory focus on correcting unhealthy habits and promoting recovery from disease through gamified content. 

The G4H theory emphasizes that the primary objective of behavioral interventions in health games must be to help users or patients achieve specific health goals. However, the theory does not standardize the methods and criteria for measuring the effectiveness of the interventions and the functional modules applied.

#### 2.2.2. Standards for Functional Design in Digital Health Applications

According to the definition of the World Health Organization (WHO), digital health refers to the use of digital technology to improve human health [45]. Among the various subcategories of digital health, digital therapeutics is an important one. The core processes of digital therapeutics include prevention, management, and treatment [46]. A digital therapeutics-based application should meet the following four requirements:

1. The application must be driven by software.
2. The therapeutic effectiveness must be supported by scientific evidence.
3. The application must provide specific intervention measures for a designated disease, with the software’s functionality serving as the core mechanism.
4. The application must be designed for patient use.

The main purpose of digital health applications is to provide daily healthcare and exercise guidance, and this study argues that the content and interaction of digital health applications should comply with basic medical-scientific safety standards and have the core objective of preventing or slowing down the occurrence and deterioration of health problems. At the same time, the functional design should be as close as possible to the functional requirements of digital therapeutics. Ideally, a multifunctional digital health application would enable users to learn healthcare exercises interactively, infer their health status from collected data, and aid family members, friends, or doctors in understanding their health better.

#### 2.2.3. GameFlow Evaluation Model

The GameFlow theoretical model is synthesized from the works of Sweetser et al. [47] and Chen [48], who analyzed the theory of flow in games. They examined intrinsic factors such as elemental conditions and background conditions that constitute the flow experience. This model integrates overall game usability and user experience factors. It assists game designers in creating game experiences from the player’s perspective across eight dimensions: attention, challenge, player skill, control, clear goals, feedback, immersion, and social interaction.

## 3. Materials and Methods

### 3.1. Gamified Motion-Sensing Health Application Framework

Currently, there is a notable absence of theoretical models for gamified health applications in the market and academic domains, particularly concerning the gamified design of cervical spine exercises. Examples of teaching and evaluating the effectiveness of cervical spine exercises with mobile applications using smartphones as the main interaction device are particularly rare.

Based on the G4H theory, the digital health application standards, and the GameFlow model, this study proposes a gamified motion-sensing health application (GMH) framework, which contains three layers.

- Conceptual Layer: Relying on the G4H theory, the core design elements of gamified neck exercise applications and their specific content in cervical spine disease interventions form the conceptual layer of the GMH framework. The detailed content is presented in Table 3, where each theoretical component is matched with a corresponding design developed in our research.

- Utility Layer: Integrating the functionality of digital health, digital healthcare, and digital therapies, the gamified health applications offer users a cohesive experience from basic health education to advanced therapeutic interventions. Accordingly, the functional design of each level in the utility tier is summarized in Table 4.

- Experience Layer: This study adopts the GameFlow model as the theoretical foundation for the Experience Layer in the GMH framework. To cultivate a positive feedback loop in user experience, gamified practice designs should incorporate an award system, feedback mechanisms, and the design of social interaction features. Table 5 summarizes how the eight factors are applied in the application.

This study has established a comprehensive GMH framework, providing a robust theoretical framework and scientific guidance for subsequent implementation.

### 3.2. Implementation of a Gamified Somatic Health Application

This study employed a high-precision optical motion capture device for skeletal animation creation and developed a gamified health application using Unity 3D technology. The application leverages the built-in Inertial Measurement Unit (IMU) of smartphones to enable somatosensory interactions. Utilizing the standard cervical spine health exercise paradigms outlined in Section 2.2, the application integrates the “looking up at the stars” action to design the corresponding interaction.

**Figure 1:**
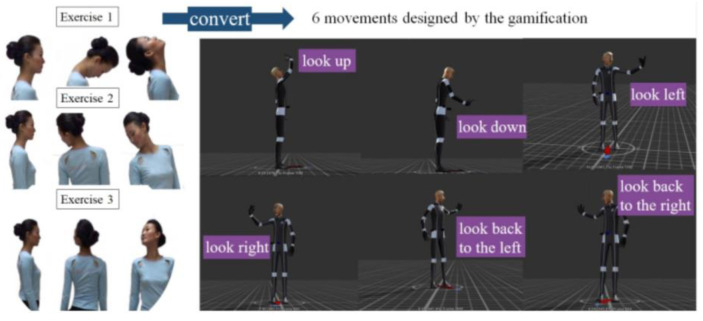
Three neck exercises and 6 movements in gamification design.

During interactive exercises, users must align their gaze with the center of the phone screen, keep their arms straight, and use arm movements to drive horizontal or vertical movements of the shoulders and neck. Figure 1 illustrates the postures of the 3D model captured by motion capture technology and the actual neck exercises.

**Figure 2:**
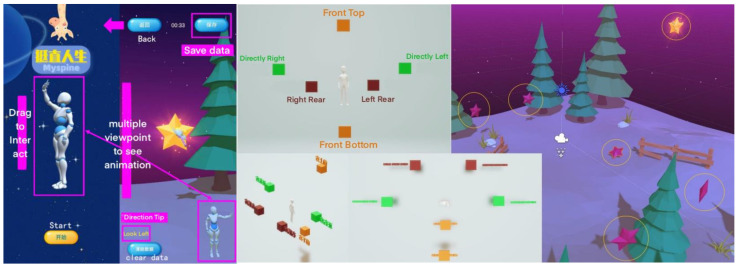
Game interaction and gameplay schema.

The main interface of the application displays a 360-degree rotatable exercise demonstration model, enabling users to observe exercise movements in detail. By clicking the “Start” button, users enter the cervical spine gamified somatosensory exercise game. In the game, users move the smartphone to align the magic wand with the star on the screen and maintain this position for 3 s until the star disappears. Following the prompts in the lower-left corner of the screen, users proceed to the next movement. This process can be repeated without level restrictions, and users may exit the game at any time. Capturing six stars completes a full exercise round. Figure 2 displays the application’s main interface and the game.

Furthermore, the study implements a reward system designed to enhance users’ sense of achievement and satisfaction. The reward mechanism provides “social currency,” enhancing user sharing and social interaction. These designs adhere to the “feedback” and “social interaction” standards of the GMH framework’s Experience Layer.

### 3.3. Experimental Setup

The study employed an “experimental study” design according to *the Lancet’s* Categories of Clinical Research framework [49], conducting a randomized control group concurrent controlled trial.

The study implemented two distinct intervention schemes: a text and video intervention approach and a gamified approach. Participant performance was assessed before and after interventions under both schemes in real-life settings. The following subsections discuss the ethical standards for participants (Section 3.3.1), the experimental setup (Section 3.3.2), and the data collection methods (Section 3.3.3).

#### 3.3.1. Participants and Ethics

The study recruited 19 university students and one community member to participate in this study. All participants had no history of serious illnesses. Ethical approval for this study was obtained from the Life Ethics Committee of the Tsinghua University Shenzhen International Graduate School. All participants read and signed the consent form. A total of 20 participants were randomly assigned in a 1:1 ratio to the experimental group and the control group. The experimental group used a gamified cervical spine exercise application, while the control group received daily cervical spine health information in text and video formats via a WeChat group.

#### 3.3.2. Setup of the Study

**Figure 3:**
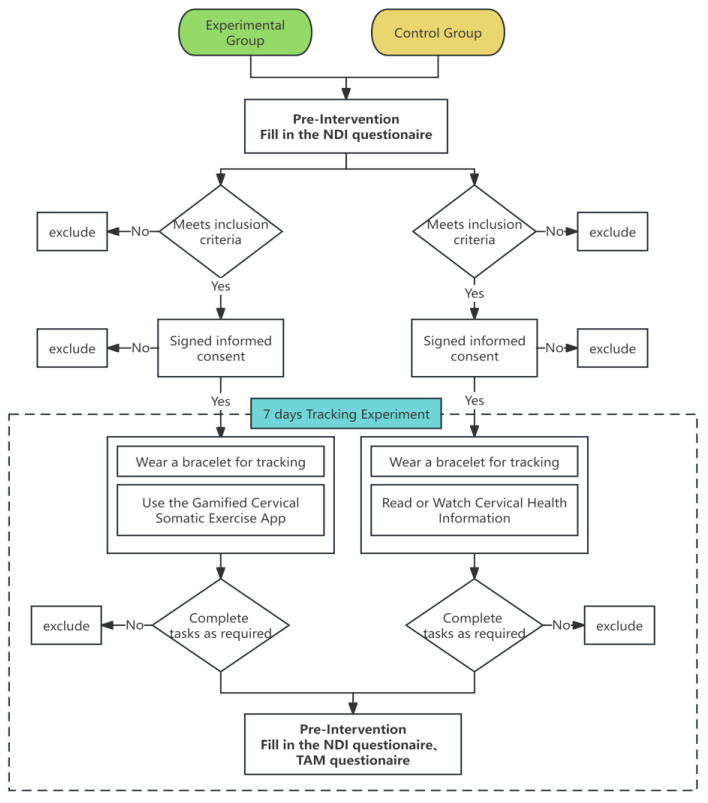
Process of the study experiment.

The specific experimental procedures were divided into three stages, as illustrated in Figure 3.

1. Pre-experiment: Both control and experimental group members filled out the NDI questionnaire (refer to Appendix A, introduced in Section 3.3.3) and recorded their step data for the four days preceding the experiment. These data served as a baseline for physical activity indicators before the intervention.
2. During the experiment: Both groups recorded their daily step data. Control group members received text and video instructions via a WeChat group. In contrast, experimental group members engaged with a gamified health application. Participants manually clicked a “Save” button within the application to store their data in the backend database.
3. Post-experiment: Both groups completed the NDI questionnaire again. Additionally, experimental group members filled out the TAM questionnaire and submitted the application data to the researchers.

#### 3.3.3. Data Collection and Processing

To obtain objective and precise activity data for experimental verification, this study provided participants with exercise bracelets to collect their movement data.

Hardware Specifications: The exercise bracelets were equipped with optical heart rate monitors and capacitive sensors to capture the participant’s heart rate data. Additionally, they included a six-axis motion sensor composed of gyroscopes and accelerometers, supplemented by GPS for location calibration, enabling the collection of high-precision step data.

Matching Software: Participants could download and install the Keep app to view basic exercise information, including step count, sleep duration, and blood oxygen saturation.

The study collected the following data:

- Exercise Duration

Each round of exercise duration was calculated based on the time difference between the disappearance of the first and the sixth star, which was then compared with clinically recommended exercise durations to validate the appropriateness of exercise lengths.

- Total Exercise Duration

The total exercise duration was calculated by multiplying the number of app openings by the duration of each exercise session.

- Startup Rate

An effective startup was recorded each time the application was opened and at least one exercise session was completed, involving interaction with all six stars. The startup rate is a key indicator of user engagement and participation.

- Step Count

The study collected participants’ step data through an online form for the four days preceding and during the experiment, measuring daily activity levels. Changes in participants’ activity levels were assessed to determine the impact of the study on raising health awareness and promoting increased daily activity.

- Neck Disability Index (NDI)

The study employed the NDI questionnaire to evaluate changes in cervical spine health status between control and experimental group members. This questionnaire encompasses five dimensions, assessing ten aspects: pain intensity, personal care (washing, dressing, etc.), lifting heavy objects, reading, headache, concentration, work, sleep, driving, and recreation. Each item on the questionnaire is scored from 0 to a maximum of 5, with higher scores indicating more severe cervical spine dysfunction.

- Technology Acceptance Model (TAM)

The TAM Questionnaire was introduced by Professor Davis in 1986, whereby the TAM helps analyze user behavior and system usage rates [16]. This study’s TAM addressed “perceived usefulness,” “perceived ease of use,” and “attitude towards using” with an 11-point Thurstone scale, ranging from “strongly disagree” (0) to “strongly agree” (10). Specific questionnaire items are included in Appendix B.

- Likert 5-Point Scale

**Figure A1:**
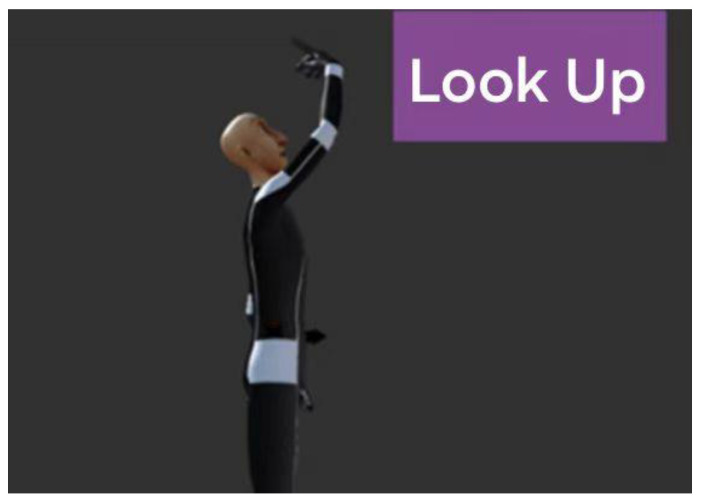
Look-up posture perception.

The Likert 5-Point Scale is a widely used psychometric tool that allows respondents to express their level of agreement or disagreement with a given statement [50]. This scale was employed in the present study for the statistical analysis of stretch perception related to six gamified cervical spine exercise movements. For detailed information on the questionnaire design, please refer to Appendix C (Figure A1).

## 4. Results

All the collected data were stored, organized, and visualized via the Microsoft Excel software application. Statistical analyses were conducted using the IBM SPSS software package.

### 4.1. Results of the Data Collected to Test H1: Gamification Method Is More Effective Than Traditional Text and Video Interventions for Neck Pain

#### Analysis of the NDI Score

In the course of analyzing the experimental data, internal consistency was evaluated. The Cronbach’s alpha coefficients for the experimental and control groups were 0.755 and 0.844, indicating a good level for the experimental group and a satisfactory level for the control group. Given the sample size was less than 50, the Shapiro–Wilk (SW) test was utilized to ensure that the data met the normal distribution assumptions necessary for subsequent statistical tests. The independent samples t-test compared the NDI scores of the experimental and control groups before and after the intervention. The results indicated statistically significant differences in the mean NDI scores of the participants between the two groups, with all *p*-values < 0.05.

Table 6 below shows the detailed individual mean scores of the Neck Pain Index for the control group before and after the experiment.

Under the health management intervention involving only health information articles and videos, the control group exhibited minimal change in the average scores for each item in the NDI over 7 days. The overall pain level slightly increased by 0.02. This indicates that the 10 participants in the control group did not experience significant changes in their perceived neck pain during the trial period.

As can be seen in Table 7, during the 7 days of using the gamified exercise application, the experimental group showed a downward trend in the average scores for 10 items in the NDI. The final average NDI score decreased from 1.54 to 1.24, a reduction of 0.30. The most significant improvement was in sleep, which decreased by 0.5, followed by pain intensity and work, each decreasing by 0.4. 

**Figure 4:**
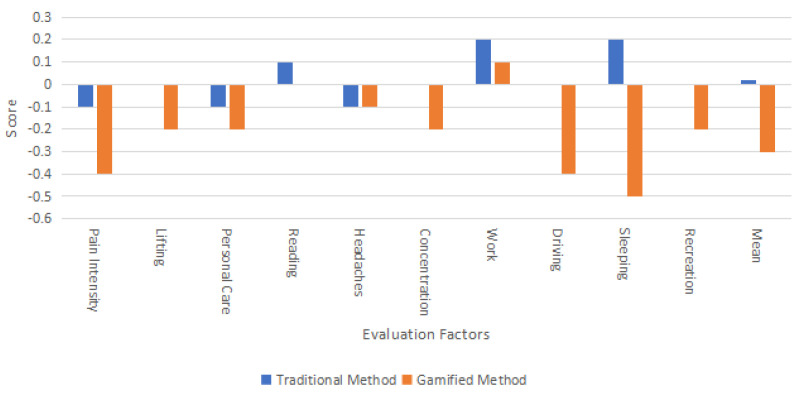
Comparison of NDI score changes between two intervention methods (for clarity, only changes in NDI score are displayed in the chart).

Overall, the gamified exercise application reduced neck pain in the experimental group, proving to be more efficacious than the traditional text and video intervention used in the control group. Figure 4 illustrates this comparison with a bar chart showing the changes in NDI scores for both groups.

### 4.2. Results of the Data Collected to Test H2: The Gamification Method Can Enhance User Compliance in Neck Pain Interventions

#### 4.2.1. Step Count Analysis

The results indicated an 8.64% decrease in the control group’s step count, while the experimental group’s step count increased by 22.66%, approaching the recommended 6000 steps per day. These findings indicate that text and video interventions did not significantly improve the control group’s step count. In contrast, the gamified exercise application approach motivated participants to increase daily activity, reduce sedentary behavior, and enhance health awareness and engagement. These results validate the hypothesis that gamification can effectively enhance user compliance.

#### 4.2.2. Exercise Duration Analysis

Among the participants who performed cervical spine exercises at least once per day, the 7-day average single exercise duration ranged between 148 and 178 s, aligning with the recommended interaction duration range proposed in Table 2. The average startup rate in the experimental group was 87.5%, and each participant opened the application an average of 6 times per day. According to the equation:

Total Exercise Duration = Number of Opens × Duration of Single Exercise,(1)

The daily total exercise duration of the participants was about 18 min. The data indicate that the frequency and duration of engagement with the gamified health application in the experimental group were significantly high.

Participants in the control group performed cervical spine exercises through text and video guidance for a shorter period each day, averaging approximately 5 min, and only persisted on an average of 5 out of 7 days. This suggests that traditional health education methods may be inadequate in maintaining engagement and effectiveness compared to gamified health applications.

#### 4.2.3. Analysis of User Technology Acceptance and Action Perception

The Cronbach’s alpha coefficient for the Technology Acceptability scale was 0.94, and for the Gamified Action User Perception scale, it was 0.823. Both coefficients were greater than 0.8, indicating the high reliability of the research data obtained from these scales.

Table 8 shows that most participants could perceive the stretching sensation in their neck and shoulders caused by rotation in different directions during the gamified cervical spine exercise. This indicates that the gamified interaction method effectively achieved the desired cervical spine exercise outcomes.

As shown in Table 9, in the “Perceived Usefulness” section of the TAM scale, participants in the experimental group mostly scored 8 or above, indicating their recognition of the gamified somatosensory application’s overall usefulness. The data on increased willingness to stand up and walk align with the increased step counts recorded by the bracelet data.

The “perceived ease of use” section data revealed statistically significant differences (*p* < 0.05), with participants in the experimental group scoring above 8. This indicates that the experimental group found the interactive program user-friendly.

As illustrated in Table 10, on the assessment of the “Intention to Use” questionnaire, overall high levels of satisfaction and willingness to continue using the program were presented.

## 5. Discussion

This study addresses the following research question: *Can a gamified neck exercise application improve the effectiveness of CNNP interventions?* We investigated this through two distinct hypotheses.

Our first hypothesis, “**H1** = *Gamification method is more effective than traditional text and video interventions for neck pain*.”, is supported by our findings through a 7-day, 20-person control experiment. The results showed that in the experimental group, which utilized a gamified neck exercise application, the NDI scores significantly decreased from 1.54 to 1.24. Although the decrease in NDI scores was modest, it was statistically substantial (*p* < 0.01). In contrast, the control group, which only provided text and video content, showed no obvious change in NDI scores before and after the intervention, indicating a lack of substantial intervention impact. However, this study has certain limitations. The average age of participants was 23, and they experienced relatively mild neck pain issues or had low awareness of neck pain, likely contributing to the lower self-reported NDI values. Nonetheless, our findings still support the validity of Hypothesis 1. Therefore, gamified applications for neck pain interventions can be more widely applied and promoted among target populations needing such treatments.

Regarding our second hypothesis, “**H2** = *The gamification method can enhance user compliance in neck pain interventions.*”, compared to the traditional text-and-video-guided approach, participants in the experimental group spent more time per session using the gamified health application. Their exercise duration met the required standards. The average daily step count increased by 22.66%. Participants also reported a noticeable sensation of cervical traction during application use, indicating a positive user experience, higher satisfaction, and a willingness to continue using the application. The dissatisfaction among those unwilling to continue was attributed to the limited variety in the gamified exercise regimen. Thus, developing more complex and diversified game mechanics is suggested. On the contrary, the control group did not demonstrate any improvements in their daily step counts or sedentary behaviors. Follow-up surveys revealed that participants in the control group generally reported increased fatigue from reading text and a degree of aversion to this method, leading to a higher likelihood of abandonment and fewer improvements in neck pain.

In the treatment of CNNP, traditional low-cost interventions such as text and video tutorials are widely used but often suffer from a lack of personalized guidance and real-time feedback, resulting in poor user adherence and outcomes. Although common hospital-based physical therapies such as acupuncture, cupping, and osteopathic manipulation provide individualized guidance and treatment protocols, the need for trained physicians and the risk of adverse events [51], as well as the high time and financial costs, have made it difficult for these therapies to be widely adopted.

Currently, there are several digital therapy products for neck pain intervention, such as the Kinect-based neck rehabilitation games developed by Schonauer C [12] and the VR games proposed by Mads Kloster [52]. However, these methods require expensive and hard-to-disseminate equipment and are often accompanied by motion sickness issues in VR [53]. The application developed in this study offers a more lightweight and universal solution, operating easily on a mobile phone. It significantly reduces treatment costs and has demonstrated high user compliance through empirical validation.

Meanwhile, this study developed a framework for gamified somatosensory health applications that feature comprehensive functions and provide excellent user experiences, addressing the shortcomings of existing theoretical models. Based on this framework, a gamified cervical spine exercise application was developed, demonstrating superior user experience and intervention effectiveness. This confirms that the framework can effectively guide the development of such applications.

To further expand the applicability of the GMH framework and the experimental design in other health domains, several challenges need to be considered:

1. Adaptation of Motion Capture Technology: Different rehabilitation exercises involve varying movements, necessitating appropriate motion capture and data processing technologies to record and analyze these movements accurately.
2. Integration of Professional Knowledge: Collaboration with experts in relevant fields is required to ensure that the exercise movements and protocols within the game are scientifically sound and safe.

Overall, these findings provide robust support for future widespread adoption of gamified health applications and will aid in refining the framework’s utility.

## 6. Conclusions and Future Work

This study validates the efficacy of the GMH framework designed to steer the development of digital health applications. By incorporating motion capture technology, the application provides users with precise motor learning references, ensuring the accuracy and safety of the exercises. Controlled experimental outcomes demonstrate that the application not only alleviates cervical pain but also boosts compliance, outperforming traditional physical therapies. Its accessibility and cost-effectiveness enhance its wide applicability, making it highly suitable for widespread use among smartphone users. The findings advocate for its integration into routine care practices, potentially extending to other rehabilitation domains to address similar health challenges.

For future research, the following recommendations are proposed to enhance the depth and breadth of investigations into gamified health applications:

1. Increase the sample size to encompass a broader range of age groups, and focus on populations at high risk of significant cervical spine issues.
2. Extend the experimental period to 4–6 weeks to explore the long-term effects.
3. Develop complex game mechanics to enrich user interactions, thereby improving the adherence and the effectiveness of the treatment.
4. Incorporate a variety of objective measurement tools, including muscle tone testers and X-ray imaging, to provide a more precise assessment of user activity levels and health status.
5. Consider using advanced computer vision technology, such as learning from a single visual observation [54], to reduce reliance on motion capture devices.

## Figures and Tables

**Table 1:** Comparative summary of existing treatments for chronic non-specific neck pain.

Treatment Type	Description
Drugs [19]	Medication such as non-steroidal anti-inflammatory drugs (NSAIDs)
Cervical Massage [20]	Uses manipulation or instruments (such as a massage hammer, massage pad, and massage chair) to stimulate specific parts of the human body
Acupuncture [21]	A traditional Chinese practice involves the insertion of fine needles into specific points on the body to balance the flow of energy.
Physical Exercise [22]	Exercise that includes neck stretching and training
Manual Therapy [23]	Delivered by specially trained physicians, using muscle techniques to relax tight muscles
Osteopathic manipulative treatment [24]	A variety of techniques applied by osteopathic physicians to manipulate the body’s musculoskeletal system
Cupping Therapy [25]	Alternative medicine technique involving suction cups placed on the skin; promotes blood flow and muscle relaxation

**Table 2:** Extracted cervical spine exercise movements for gamification.

Researcher	Description of the Intervention Method	Number of Participants
Bobos et al., 2016 [28]	Move the head backward in a sitting position; Move the head forward in a sitting position; Move the head to the left in a sitting position; Move the head to the right in a sitting position.	67
Chung et al., 2018 [43]	Isometric neck flexion/extension/rotation/lateral flexion in a seated position using hands as counter resistance; 3 workouts per week; perform 10–15 repetitions of each workout with 10 s of holding and 15 s of rest.	41
Yildiz et al., 2017 [30]	Cervical spine extension (anterior and posterior recession exercises using a towel/elastic band as a resistance hold).	30
Shiravi et al., 2019 [34]	Press the top of your head (hold your hand on your forehead); retraction and outward rotation.	135

**Table 3:** Content design of the Conceptual Layer of GMH.

Components	Corresponding Design
Gamified interaction methods	Using the gyroscope on the phone helps users to conduct gamified motion-sensing interaction.
Behavioral determinants	Transform the habit of using smartphones into a motion-sensing interaction for gamified exercise.
Behavioral intervention	Help users reduce sedentary behavior through gamified interactive content.
Identification of Antecedents of health Problems	Help users reduce their resistance and fear in learning neck exercises. These exercises can be completed using only one mobile phone, dispelling concerns about space and equipment restrictions.
Achievement of health goals	Enhancing users’ awareness of cervical spine care, imparting exercise skills, preventing cervical spondylosis, and reducing sedentary behavior.

**Table 4:** Content design of the Utility Layer of GMH.

Factor	Major Functions and Design
Digital health	Provide information on cervical spine healthcare teaching content
Digital healthcare	Infer the user’s cervical spine health status based on the user’s cervical spine movement angle information
Digital therapeutics	Provide cervical spine health exercise program with clinical evidence to improve users’ cervical spine health condition

**Table 5:** Content design of the Experience Layer of GMH.

Factor	Corresponding Design
Attention	“Find the Stars” interactive content design
Challenge	Learn how to perform neck exercises and the timings of the exercises
Player skill	High-precision digital model demonstration animation, gamified interactive guidelines
Control	Full-angle motion-sensing exploration in virtual space
Clear goal	Science-based star position setting and interaction sequence arrangement in virtual space
Feedback	Gamified visual feedback and sound feedback
Immersion	Realistic physical settings and scenarios
Social interaction	Reasonable virtual asset rewards

**Table 6:** Comparison between pre/post-intervention in the mean NDI Scores (control group *n* = 10).

Question	Pre-Intervention	Post-Intervention	Change
Pain Intensity	2.2	2.1	−0.1
Lifting	1.9	1.9	/
Personal Care	0.5	0.4	−0.1
Reading	1.2	1.3	+0.1
Headaches	1.6	1.5	−0.1
Concentration	1.8	1.8	/
Work	2.0	2.2	+0.2
Driving	1.4	1.4	/
Sleeping	1.5	1.7	+0.2
Recreation	0.7	0.7	/
Mean	1.48	1.50	+0.02

**Table 7:** Comparison between pre/post-intervention in the mean NDI Scores (experimental group *n* = 10).

Question	Pre-Intervention	Post-Intervention	Change
Pain Intensity	2.1	1.7	−0.4
Lifting	2.0	1.8	−0.2
Personal Care	0.6	0.4	−0.2
Reading	1.3	1.3	/
Headaches	1.5	1.4	−0.1
Concentration	2.0	1.8	−0.2
Work	1.9	1.9	+0.1
Driving	1.6	1.2	−0.4
Sleeping	1.8	1.3	−0.5
Recreation	0.6	0.4	−0.2
Mean	1.54	1.24	−0.3

**Table 8:** Gamified movement user perception ratings (N = 10).

Question	Min	Max	Mean	SD	*p* Value
What is your level of cervical stretching sensation when you are doing a head-up maneuver?	3.000	5.000	4.100	0.738	*p* < 0.001
What is your level of cervical stretching sensation when you are doing a head-down maneuver?	2.000	5.000	3.400	0.843	*p* < 0.001
What is your level of cervical stretching sensation when you look to the left?	4.000	5.000	4.300	0.483	*p* < 0.001
What is your level of cervical stretching sensation when you look to the right?	4.000	5.000	4.300	0.483	*p* < 0.001
What is your level of cervical stretching sensation when you do the left backward-looking maneuver?	3.000	5.000	4.400	0.843	*p* < 0.001
What is your level of cervical stretching sensation when you do the right backward-looking maneuver?	3.000	5.000	4.500	0.707	*p* < 0.001

**Table 9:** One-sample *t*-test for TAM questionnaire “perceived usefulness” part (N = 10).

Question	Min	Max	Mean	SD	*p* Value
Exercising with the app helps me relax my cervical spine.	6.000	11.000	9.200	1.398	*p* < 0.001
Exercising with the app helps me reduce my sedentary time.	4.000	11.000	8.500	2.068	*p* < 0.001
Exercising with the app improves my willingness to get up and walk.	4.000	11.000	8.600	1.955	*p* < 0.001
Exercising with the app helps me be more aware of my cervical spine health.	6.000	11.000	8.900	1.663	*p* < 0.001
Exercising with the app is useful.	6.000	11.000	9.100	1.595	*p* < 0.001

**Table 10:** One-Sample *t*-test for TAM questionnaire “Intention to Use” part (N = 10).

Question	Min	Max	Mean	SD	*p* Value
I like to use the “Find the Stars” interactive workout in the app.	8.000	11.000	9.400	0.843	*p* < 0.001
I’m positive about the interactive exercise function of the app.	8.000	11.000	9.500	0.850	*p* < 0.001
I think the app’s interactive workout design is an innovative feature.	8.000	11.000	9.700	0.949	*p* < 0.001
If I’m using the app, I’ll continue to use the Somatic Interaction feature to relax my cervical spine	5.000	11.000	9.200	1.687	*p* < 0.001

## Data Availability

All relevant data are within the manuscript.

## Appendix A. Neck Disability Index (NDI) Questionnaire

Name:

Date:

Section 1: Pain Intensity

☐ I have no pain at the moment

☐ The pain is very mild at the moment

☐ The pain is moderate at the moment

☐ The pain is fairly severe at the moment

☐ The pain is very severe at the moment

☐ The pain is the worst imaginable at the moment

Section 2: Personal Care (Washing, Dressing, etc.)

☐ I can look after myself normally without causing extra pain

☐ I can look after myself normally but it causes extra pain

☐ It is painful to look after myself and I am slow and careful

☐ I need some help but can manage most of my personal care

☐ I need help every day in most aspects of self-care

☐ I do not get dressed, I wash with difficulty and stay in bed

Section 3: Lifting

☐ I can lift heavy weights without extra pain

☐ I can lift heavy weights but it gives extra pain

☐ Pain prevents me from lifting heavy weights off the floor, but I can manage if they are conveniently placed, for example, on a table

☐ Pain prevents me from lifting heavy weights but I can manage light to medium weights if they are conveniently positioned

☐ I can only lift very light weights

☐ I cannot lift or carry anything

Section 4: Reading

☐ I can read as much as I want to with no pain in my neck

☐ I can read as much as I want to with slight pain in my neck

☐ I can read as much as I want with moderate pain in my neck

☐ I can’t read as much as I want because of moderate pain in my neck

☐ I can hardly read at all because of severe pain in my neck

☐ I cannot read at all

Section 5: Headaches

☐ I have no headaches at all

☐ I have slight headaches, which come infrequently

☐ I have moderate headaches, which come infrequently

☐ I have moderate headaches, which come frequently

☐ I have severe headaches, which come frequently

☐ I have headaches almost all the time

Section 6: Concentration

☐ I can concentrate fully when I want to with no difficulty

☐ I can concentrate fully when I want to with slight difficulty

☐ I have a fair degree of difficulty concentrating when I want to

☐ I have a lot of difficulty in concentrating when I want to

☐ I have a great deal of difficulty in concentrating when I want to

☐ I cannot concentrate at all

## Appendix B

Upright Life Interaction Somatic Exercise Application Using Feedback Questionnaire

Technology Acceptance Model (TAM) Questionnaire											
	0	1	2	3	4	5	6	7	8	9	10
“Perceived usefulness”											
Exercising with the app helps me relax my cervical spine.											
Exercising with the app helps me reduce my sedentary time.											
Exercising with the app helps me improve my willingness to get up and walk.											
Exercising with the app helps me be more aware of my cervical spine health.											
Exercising with the app is useful.											
Perceived Ease of Use											
The app’s operating instructions are clear.											
The app is easy to interact with.											
I’m satisfied with the interactive workout feature using the app.											
I make very few operator errors when performing the Find the Stars workout interaction.											
I think the app operation works well with my movement for virtual space exploration.											
I think for the purpose of exercising the cervical spine, the star position makes sense in the app.											
I rarely have unexpected problems when I use the app.											
Intent to use											
I like to use the “Find the Stars” interactive workout in the app.											
I’m positive about the interactive exercise function of the app.											
I think the app’s interactive workout design is an innovative feature.											
If I’m using the app, I’ll continue to use the Somatic Interaction feature to relax my cervical spine											

## Appendix C

Stretch perception of the gamified cervical spine exercise movements (excerpt).

What is the level of cervical spine stretching you feel when you are doing a headlift?

○ Very inconspicuous ○ Not obvious ○ Moderate ○ Obvious ○ Very obvious
